# Making psychiatry a household word

**DOI:** 10.4103/0019-5545.31513

**Published:** 2007

**Authors:** I. R. S. Reddy

**Affiliations:** Director, Vijayawada Institute of Mental Health and Neurosciences (VIMHANS), Vijayawada, AP, India

Respected chair persons and members of the Indian Psychiatric Society (IPS), I am extremely happy and privileged to be here at the 59^th^ Annual Conference, before an enlightened gathering to preside over the prestigious IPS. It is a rare honour and it shall be my endeavour to prove myself worthy of being chosen for this prestigious and highly coveted honour among the psychiatrists. I know my limitations but I am also aware of the great role that can be played by our society and I make bold to place before you many of my experiences in the field to exhort my fellow psychiatrists that we have a great responsibility ahead. From what I have observed during these years of my active practice, I have no doubt in my mind that the psychiatry as a profession is slowly gaining ground and in the years to come it will play an effective role in disabusing the minds of the public of their wrong and ill conceived notions. I shall endeavour my best with the cooperation and support of all my fellow psychiatrists. There were quite a few topics that I short listed for my presidential address. Finally, I zeroed in on this topic titled “MAKING PSYCHIATRY A HOUSEHOLD WORD” as I feel the art and science of psychiatry has a great deal to offer society apart from treating the “crazy people”. To make that contribution, psychiatrists must continue to tackle society's most pressing problems and also raise the visibility of psychiatry and its perceived relevance to solving a wide range of personal, social and family problems. The image of psychiatry has been tarnished in the eyes of common man, thanks to the battering that the profession has received at the hands of the media, not to mention the apathy of the policy makers.

Here is an example to prove my point. At the recently held conference at Jaipur, I was walking outside the convention center when I heard two young women talking to each other. One said to the other: “My God!!!…. He's so weird! He really should see one of those psychiatrists who are walking around here.” “That's it!” I thought. “People think you have to be ‘weird’ to see a psychiatrist.” The public tends to view psychiatrists narrowly, associating us chiefly with our expertise in mental illness. In reality, psychiatrists can promote coping and wellness in addition to diagnosing and treating mental illness. Many people today have significant stress in their lives and we all undergo crises and life-stage transitions as a normal part of life. Psychiatrists can help people assess how they are coping with current stresses and develop new skills and strategies. A lot of people find that their stress levels have increased in this era of wars, terrorism and natural disasters. We, as psychiatrists, could do much more to prevent problems by helping people learn to cope and build their resilience. I wondered what it would take to normalize our public image, that set me thinking and I looked into dentistry, which changed the public image of its profession in the 1980s when it teamed up with Colgate toothpaste in a public education campaign that promoted the idea of the dental check-up. This campaign led to major changes not only in the public image of dentistry but also in the very nature and public impact of dental practice. Dentistry moved from a focus on restorative dentistry to an emphasis on preventive dentistry. We have all witnessed the success of this transition. People no longer wait for a toothache to visit the dentist and employers routinely include preventive examinations and cleanings as a dental benefit. I am envisaging a day when people similarly take appointments for a “psychological check-up” akin to a general health check -up and when that day comes one can rest assured that the community has truly embraced our profession. At these check-ups they could address such matters as their stress level, their relationships, how they are caring for their children and ageing parents and health basics such as diet, nutrition, sleep and exercise.

In my 30 years of practising psychiatry, I have had a unique vantage point from which to both observe and reflect on, the process whereby psychiatry responds to urgent societal needs and how these needs thus influence the evolution of psychiatry. As I see it, the scope of psychiatric practice is expanding and diversifying into new areas. In short, the stage is set for the public's as well as policymakers and health care payers' full embrace of our field and for the true integration of psychiatry into health care, Only if we make them aware of our scientific knowledge and professional skills.

## DISABILITY AND MENTAL ILLNESS

The burden of mental illness on health and productivity throughout the world has long been profoundly underestimated. Data developed by the massive Global Burden of Disease study conducted by the World Health Organization, the World Bank and Harvard University, reveal that mental illness, including suicide, accounts for over 15 percent of the burden of disease in established market economies, such as the United States. This is more than the disease burden caused by all cancers.[1]

This Global Burden of Disease study developed a single measure to allow comparison of the burden of disease across many different disease conditions by including both death and disability. This measure was called Disability Adjusted Life Years (DALYs). DALYs measure lost years of healthy life regardless of whether the years were lost to premature death or disability. The disability component of this measure is weighted for severity of the disability. For example, disability caused by major depression was found to be equivalent to blindness or paraplegia whereas active psychosis seen in schizophrenia produces disability equal to quadriplegia. The World Health Organization's Global Burden of Disease study reported that mental disorders comprise four of the top five sources of premature death and disability in 15-44 year olds in the Western world.

Using the DALYs measure, major depression ranked second only to ischemic heart disease in magnitude of disease burden in established market economies. Schizophrenia, bipolar disorder, obsessive-compulsive disorder, panic disorder and post-traumatic stress disorder also contributed significantly to the total burden of illness attributable to mental disorders. The projections show that with the aging of the world population and the conquest of infectious diseases, psychiatric and neurological conditions could increase their share of the total global disease burden by almost half, from 10.5 percent of the total burden to almost 15 percent in 2020. Major depression is the leading cause of disability (measured by the number of years lived with a disabling condition) worldwide among persons age 5 and older. For women throughout the world as well as those in established market economies, depression is the leading cause of DALYs. In established market economies, schizophrenia and bipolar disorder are also among the top 10 causes of DALYs for women.

The above stated facts, hopefully will be an eye-opener for all the concerned parties. From our side, we should do all that we can in whichever small way to reduce this enormous disease burden. My presidential address, I hope, will serve as an initiative to formulate ways and means to achieve this objective.

## IMPEDIMENTS IN MAKING PSYCHIATRY A HOUSEHOLD WORD

There have been a lot of impediments in making psychiatry a household word. The main culprits are the age old concept of mind-body dualism and lack of integration of mental health care into primary health care, stigma, psychiatrists themselves, the apathy of the policy makers, failure of Consultation - Liaison psychiatry and media. I will make an attempt to briefly detail what resulted in these hindrances and some plausible solutions to overcome them.

## MIND-BODY DUALISM

The earlier concepts of health glorifying mind-body dualism are bankrupt now and mind-body dualism has an enormous negative impact on our health care system. Because of it, our health care system does not systematically attend to the many psychological risk factors for both morbidity and mortality and it virtually ignores the psychosocial pathways that lead to unnecessary utilization of medical and surgical services. In addition, the psychological impact of having a medical illness is not well addressed by the health care system, nor is the fact that many people suffering from a physical illness have comorbid psychological illness. Finally, the lion's share of mental health problems are treated, ineffectively, by primary care providers.

Let's take a look at some of the evidence:

1. Seven of the top health risk factors are behavioral (tobacco use, alcohol abuse, poor diet, injuries, suicide, violence and unsafe sex).
2. Seven of the nine leading causes of death have significant behavioral components.
3. At least 50% (and maybe as much as 75%) of all visits to primary care medical personnel are for problems with a psychological origin (including those who present with frank mental health problems and those who somaticize), or for problems with a psychological component (including those with unhealthy lifestyle habits such as smoking, those with chronic illnesses and those with medical compliance issues).
4. Stated another way, one study found that less than 16% of somatic complaints had and identifiable organic cause.
5. A large number of studies have demonstrated that providing behavioral health care reduces the utilization of medical and surgical care.
6. The vast majority of people receiving mental health treatment are cared for by medical professionals with minimal specific training in mental health.

Moreover, there is a growing body of empirical evidence supporting the effectiveness of psychological interventions in ameliorating a wide range of physical health problems, including both acute and chronic disease affecting literally every organ system and encompassing pediatric, adult and geriatric populations. In addition to being clinically effective, these interventions are dramatically less expensive than alternative somatic interventions across a wide variety of illnesses and disorders, including cardiovascular disease, hypertension, diabetes, neoplasms and traumatic brain injury.

## INTEGRATION INTO PRIMARY HEALTH CARE

Evidence exists that basic mental health services generally can be managed in primary health care organizations with considerable cost savings and without detrimental effects on health, but it is less clear whether this is true of services for persons with severe and persistent mental illness. There is a long history of discrimination in insurance coverage for persons with mental disorders that would be corrected substantially within a single system of care that seeks to integrate necessary medical and mental health services. Many mental health problems are typically first seen as somatic presenting complaints and thus the general health sector is a natural entry point for appropriately identifying and treating mental health problems. Many patients also continue to find care for psychiatric problems more acceptable when provided by their primary care doctors. The close connection between physical and mental health care in an integrated system also provides a higher likelihood that common comorbidities between physical and mental disorders will be addressed and that improved management and communication among caregivers will reduce incompatible treatment orders and other common errors characteristic of fragmented systems of care.

The concept of integration, however, begs a number of crucial issues that are at the core of health care reform. Among them are the relationships between acute interventions and long-term care. A significant concern is that nonmedical alternatives may not be amply available in a highly medicalized system of care. As medical practitioners are pressured to be more efficient and productive, they have less time to devote to each patient and less opportunity to know the patient and to understand the complex types of problems that affect patients' functioning. Because patients with chronic conditions are commonly time consuming and costly due to their disabilities and needs, they will not be preferred clients. Persons with disabling mental illness will have to compete with other clients who may appear more attractive and easier to deal with by practitioners already working under time constraints and other stresses.

Each of these problems has possible solutions, but they are not easily achieved. Generalists can be educated to better recognize, manage and appropriately triage patients with mental disorders, but experience suggests this is a long-term and challenging task. Efforts can be made to increase the capacity of health care plans to manage serious disabling illness, but this long-term agenda must compete with many other service demands in an environment of new and increasing practice opportunities constrained by economic limits. Integration of public and private sectors is yet another novel but effective tool in achieving this objective.

Overall, the research evidence suggests that basic mental health services for less severely ill patients can be integrated into the general health sector under a broadly defined benefit and that considerable cost savings are possible without detrimental effects on health, at least for the ordinary patient. The evidence is less clear for persons with more severe and persistent disorders.

## IMAGE OF PSYCHIATRISTS - A HURDLE?

Psychiatrists should have been seen as precious and as an important resource as they are so few in numbers. In India, there are only about 3500 psychiatrists for a population of over 1080 million. Number of psychiatrists required (equivalent to the Australian rate of 13 per 100,000 population) is 140,400 which is still quite far away. Psychiatrists are seen in negative light by the community. Some of the reasons have been outlined below.

- They are seen as disease and not health-oriented (negative connotation).
- They are seen as medicine-oriented (sometimes being seen as ‘pills pushers’, ‘sedative prescribers’ etc.).
- They are seen as passing a judgment on the most important master organ in the body (mind/brain).
- They are seen as having the power to judge others as ‘mad’, not in control and thus could potentially deprive them of their rights and indeed being a human being.
- They are seen as making a judgment without any ‘specific proof’ like a diagnostic tool/investigation to ‘prove their view point’.
- They are seen as not scientific (different psychiatrists may differ in diagnoses and management plans).
- They are seen as giving torture producing treatments (ECT, aversive shocks etc.).
- They are seen as only ‘treating symptoms’ and not ‘curing the root cause’.

These perceptions have been built up over time and other mental health professionals, quacks, practitioners of other forms of medicine and healer of various kinds including faith healers have contributed to these perceptions. Exploitation of human suffering by unqualified, self proclaimed psychologists, self help Gurus, personality trainers has deteriorated matters further. The bewildering information explosion of diverse kinds and points of view has made it worse, specially uncontrolled information from the web. Sometimes, the attitudes of psychiatrists themselves, the inability to formulate clear cut guidelines and not shifting the focus from negative to positive and not creating enough awareness about our specialty, have made it worse.

We speak of the impending endemic of mental illnesses and disease in the future but are not doing anything to prevent it. We speak of inculcating positive mental health but like experts do not give any guidelines or directions. Worse still, we do not have adequate policies, directions or discussions for mental health at the governmental, professional organizations or individual level. We speak of well being while keep dissecting and debating on illnesses.

The media and awareness campaigns do not have psychiatrists as the team leaders but are excluded due to our own discomfort. We would be willing to give culturally alien explanations of causations and treatment modalities for illnesses but not comfortable in looking at the culturally accepted, cost effective, easy to adopt preventive and health promotive strategies.

We can be leaders if we are willing to lead. The community of which media is a part would be willing to allow us to lead if we have comprehensive plans for promotive, preventive, intervention and rehabilitative strategies which are holistic, cost effective and culturally relevant. We would have to join hands with other trained professionals and community leaders along with media to create long term solutions for the entire population rather than look at a narrow segment of only those who have become extremely dysfunctional and ill. We need to be visionaries for the mental health needs of the entire communities and not only the service providers for sick individuals. It is only then that psychiatrists in particular and psychiatry in general would receive its rightful place and become a “Household Word”

## CONSULTATION - LIAISON PSYCHIATRY

Some of the obstacles to liaison with non-psychiatrists are outlined and methods are suggested for eliciting the cooperation and enthusiasm required for effective patient care. Often, too, there is a disinclination on the part of some non-psychiatric physicians to acknowledge the place of emotional factors in the treatment of the whole person. This outlook may be related to personality; certain physicians prefer clear-cut and readily identifiable conditions that are easy to observe and agree upon following laboratory or clinical tests. Another obstacle to successful liaison is that mental health workers have long had a reputation for maladjustment. The reputation is, or at least was in the past, partly justified, because many people sought the field in order to solve their own psychological problems. Recently, it seems less true, although hard facts and measurements confirming such an assertion are difficult to find. The consultation-liaison literature frequently recommends that chart notes be practical and avoid the use of technical psychiatric terms. The psychiatrist's presence on rounds of non-psychiatric services or at conferences, in many cases, is met with hostility and suspicion. One fear expressed by many specialists, including psychiatrists, is that a consultant will come in vigorously swinging his elbows, declaring that the patient has a more serious disorder than the one diagnosed and that the person whose service he is currently on should relinquish the patient for psychiatric treatment. If the physician agrees, the patient gets moved to another service. If this is not agreed to, an interpersonal difficulty arises between the two care givers. This obstacle is closely related to the fear of criticism if the consultant lacks psychiatric knowledge. The liaison psychiatrist should occasionally remind himself that he, too, may lack expertise outside his specialty. Mistrust of and uneasiness with psychiatrists is a difficult problem that must be worked out over a period of time. Perhaps the most positive approach here is to make low-key but useful comments when called upon for an opinion. This may reduce any threat felt by the referring physician and allow him to pick up suggestions in the future. Some psychiatrists have a reputation for diagnosing non-patients as mentally ill. Frequently non-psychiatric physicians fear or dislike the possibility that they may be suggested to have some psychiatric problems of their own. The fear is not unfounded, as many psychiatrists and other mental health workers do, in fact, make diagnoses of fellow professionals, sometimes telling them directly or telling their co-workers. A general way to avoid many of these obstacles is for the consultant to be cautious in his approach and to recognize the primacy of other physicians and medical specialties. While clearly stating his own conclusions and suggestions, he should realize that the consultation is a conjoint effort in which the other physician will have the final say. The difficulty of dealing with psychological problems in patients with additional physical illnesses should be acknowledged and not minimized. The liaison psychiatrist should not be dramatic or suggest treatment or psychodynamics that will be regarded as very novel by the non-psychiatrist. He should also be aware of the difficulties of the non-psychiatrist's job and put forth suggestions tentatively, rather than using a take it or leave it approach. I have found that liaison with other professionals works better when I am known by name and face. When acquaintance does not occur through routine medical channels, the psychiatrist can find other ways to make himself known to the medical staff. He should cooperate with interdepartmental committees and take the business of these committees seriously. It is also useful to attend medical staff functions. The exchange of knowledge required of one another by psychiatrists and non-psychiatrists can occur during talks before such functions and can lead to the psychiatrist's becoming known. These gatherings also provide the psychiatrist with an opportunity to connect the names and faces of his non-psychiatric colleagues with their specialties and interests.

## STIGMA AND MENTAL HEALTH

Stigmatization of people with mental disorders has persisted throughout history. It is manifested by bias, distrust, stereotyping, fear, embarrassment, anger and/or avoidance. Stigma leads others to avoid living, socializing or working with, renting to, or employing people with mental disorders, especially severe disorders such as schizophrenia.[2,3] It reduces patients' access to resources and opportunities (e.g., housing, jobs) and leads to low self-esteem, isolation and hopelessness. It deters the public from seeking and wanting to pay for, care. In its most overt and egregious form, stigma results in outright discrimination and abuse. More tragically, it deprives people of their dignity and interferes with their full participation in society. Explanations for stigma stem, in part, from the misguided split between mind and body first proposed by Descartes. Another source of stigma lies in the 19^th^-century separation of the mental health treatment system in the United States from the mainstream of health. These historical influences exert an often immediate influence on perceptions and behaviors in the modern world.

In the 1950s, the public viewed mental illness as a stigmatized condition and displayed an unscientific understanding of mental illness. Survey respondents typically were not able to identify individuals as “mentally ill” when presented with vignettes of individuals who would have been said to be mentally ill according to the professional standards of the day. The public was not particularly skilled at distinguishing mental illness from ordinary unhappiness and worry and tended to see only extreme forms of behavior-namely psychosis—as mental illness. Mental illness carried great social stigma, especially linked with fear of unpredictable and violent behavior.

By 1996, a modern survey revealed that Americans had achieved greater scientific understanding of mental illness. But the increases in knowledge did not defuse social stigma.[4] Yet, in comparison with the 1950s, the public's perception of mental illness more frequently incorporated violent behavior.[4] This was primarily true among those who defined mental illness to include psychosis (a view held by about one-third of the entire sample). The 1996 survey also probed how perceptions of those with mental illness varied by diagnosis. The public was more likely to consider an individual with schizophrenia as having mental illness than an individual with depression.

This finding begs yet another question: Are people with mental disorders truly more violent? Research supports some public concerns, but the overall likelihood of violence is low. The greatest risk of violence is from those who have dual diagnoses, i.e., individuals who have a mental disorder as well as a substance abuse disorder.[5] There is a small elevation in risk of violence from individuals with severe mental disorders (e.g., psychosis), especially if they are noncompliant with their medication.[5] Yet the risk of violence is much less for a stranger than for a family member or person who is known to the person with mental illness.[5] In fact, there is very little risk of violence or harm to a stranger from casual contact with an individual who has a mental disorder. Because the average person is ill-equipped to judge whether someone who is behaving erratically has any of these disorders, alone or in combination, the natural tendency is to be wary. Yet, to put this all in perspective, the overall contribution of mental disorders to the total level of violence in society is exceptionally small.[6]

Because most people should have little reason to fear violence from those with mental illness, even in its most severe forms, why is fear of violence so entrenched? Most speculations focus on media coverage and deinstitutionalization.[6] One series of surveys found that selective media reporting reinforced the public's stereotypes linking violence and mental illness and encouraged people to distance themselves from those with mental disorders.

Some of the common stereotypes associated with patients suffering from psychiatric disorders are listed below:

**Stereotypes of mental illness**

Psychokiller / maniac

Indulgent, libidinous

Pathetic sad characters

Figures of fun

### Dishonest excuse: Hiding behind ‘psychobabble’ of doctors

It is not just that psychiatry has a shameful history in its contributions to modern-day misconceptions about mental illness, but that it has also failed to address its current deficiencies. None of the standard British psychiatry textbooks cites “stigma” in their indices. There is a dearth of *psychiatric* research on stigma and discrimination and a perennial resistance to rocking the stigma boat. Listed below are some early incorrect ideologies proposed in psychiatry, which still are fresh in peoples' minds and contribute a great deal to the mistrust in psychiatry.

### A HISTORY OF DUMB IDEAS IN PSYCHIATRY

Moon (lunatic) and womb (hysteria) theories

Technique of persuasion (brain wash)

Epileptic personalities

Mental and moral defectives

Eugenics (Ernst Rudin)

Insulin coma treatment

Frontal lobotomy

Momism, schizophrenogenic mothers, schism and skew families

Treatments for homosexuality.

### Stigma and seeking help for mental disorders

Nearly two-thirds of all people with diagnosable mental disorders do not seek treatment.[7,8] Stigma surrounding the receipt of mental health treatment is among the many barriers that discourage people from seeking treatment. Concern about stigma appears to be heightened in rural areas in relation to larger towns or cities.

### Stigma and paying for mental disorder treatment

Another manifestation of stigma is reflected in the public's reluctance to pay for mental health services. Public willingness to pay for mental health treatment, particularly through insurance premiums or taxes, has been assessed largely through public opinion polls held in western countries. Members of the public report a greater willingness to pay for insurance coverage for individuals with severe mental disorders, such as schizophrenia and depression, rather than for less severe conditions such as worry and unhappiness.[9] While the public generally appears to support paying for treatment, its support diminishes upon the realization that higher taxes or premiums would be necessary.[9] In the lexicon of survey research, the willingness to pay for mental illness treatment services is considered to be “soft.” The public generally ranks insurance coverage for mental disorders below that for somatic disorders.[9]

## REDUCING STIGMA

There is no simple or single panacea to eliminate the stigma associated with mental illness. Stigma was expected to abate with increased knowledge of mental illness, but just the opposite occurred: stigma in some ways intensified over the past 40 years even though understanding improved. Knowledge of mental illness by itself appears insufficient to dispel stigma.[4] Broader knowledge may be warranted, especially to redress public fears.[2] Research is beginning to demonstrate that negative perceptions about severe mental illness can be lowered by furnishing empirically based information on the association between violence and severe mental illness.[2] Overall approaches to stigma reduction involve programs of advocacy, public education and contact with persons with mental illness through schools and other societal institutions.[3]

Another way to eliminate stigma is to find causes and effective treatments for mental disorders. History suggests this to be true. Neurosyphilis (General paresis of Insane) and dementia due to pellagra are illustrative of mental disorders for which stigma has receded. The discoveries of an infectious etiology and of penicillin led to the virtual elimination of neurosyphilis. Similarly, when pellagra was traced to a nutrient deficiency and nutritional supplementation with niacin was introduced, the condition was eventually eradicated in the developed world. Pellagra's victims with delirium had been placed in mental hospitals early in the 20^th^ century before its etiology was clarified. Although no one has documented directly the reduction of public stigma toward these conditions over the early and later parts of this century, disease eradication through widespread acceptance of treatment (and its cost) offers indirect proof.

Ironically, these examples also illustrate a more unsettling consequence: that the mental health field was adversely affected when causes and treatments were identified. As advances were achieved, each condition was transferred from the mental health field to another medical specialty.[10] For instance, dominion over neurosyphilis (General paresis of Insane) was moved to dermatology, internal medicine and neurology upon advances in etiology and treatment. Dominion over hormone-related mental disorders (myxoedema etc.) was moved to endocrinology under similar circumstances. The consequence of this transformation, according to historian Gerald Grob, is that “The mental health field became over the years the repository for mental disorders whose etiology was unknown. This left the mental health field “vulnerable” to accusations by their medical brethren that psychiatry was not part of medicine and that psychiatric practice rested on vague hypotheses and imaginary psychodynamic formulations”.[10]

These historical examples signify that stigma dissipates for individual disorders once advances render them less disabling, infectious, or disfiguring. Yet the stigma surrounding other mental disorders not only persists but may be inadvertently reinforced by leaving to mental health care only those behavioral conditions without known causes or cures. To point this out is not intended to imply that advances in mental health should be halted; rather, advances should be nurtured and heralded. The purpose here is to explain some of the historical origins of the chasm between the general health and mental health fields.

Research that will continue to yield increasingly effective treatments for mental disorders promises to be an effective antidote. When people understand that mental disorders are not the result of moral failings or limited will power, but are legitimate illnesses that are responsive to specific treatments, much of the negative stereotyping may dissipate. Still, fresh approaches to disseminate research information and, thus, to counter stigma need to be developed and evaluated. Social science research has much to contribute to the development and evaluation of anti-stigma programs.[3] As stigma abates, a transformation in public attitudes should occur. People should become eager to seek care. They should become more willing to absorb its cost. And, most importantly, they should become far more receptive to the messages that are the subtext of this report: mental health and mental illness are part of the mainstream of health and they are a concern for all people.

Another possible remedy to stigma would be the introduction of the term “psychophobic” to describe any individual who continues to hold prejudicial attitudes about mental illness regardless of rational contrary evidence. Despite inevitable objections from some, the rise of “politically correct” language has been a key factor in the success of campaigns opposing discrimination based on gender, age, religion, colour, size and physical disability.[11]

## MEDIA AND MENTAL HEALTH

Doctors and medical profession have mostly been shown in good light in the media. However, psychiatry and psychiatrists have not been so fortunate. More often than not, the profession has been pounded by media. Mental illness has always been the substrate for comedy, sex, violence and crime, which inturn strengthens the various misconceptions in the minds of the public about mental illness and thereby increases the stigma. Leave alone, the old movies, even some of the recent Hindi movies like “Kyon Ki” have portrayed psychiatry in extremely derogatory manner. Psychiatrists are shown as punitive house keepers who give “Current Shocks” and lobotomies for the most trivial reasons. Hollywood movies are not far behind in their ignorant portrayal of mental illness. Most movie makers depict illnesses like split personality (Dissociative Identity Disorder) as Schizophrenia as if there is not enough confusion already in the minds of lay public. The print media is not far behind in its shoddy representation of psychiatry and psychiatrists. I read out an editorial by the noted journalist Vir Sanghvi which appeared in the Hindustan Times on September 15, 2006. “By the way, even though the WHO no longer lists homosexuality as a disease, the Indian Psychiatric Association continues to do so. Think of that and consider it a measure of the sophistication of the profession of psychiatry in India if you ever need to consult a shrink.”[12] This kind of publicity will scare even the most educated from seeking any kind of psychiatric help. Of late, there have been some excellent depictions of mental illness, like the blockbuster Tamil movie ‘Chandramukhi’ and ‘Annian’, both of which have been dubbed into the other regional languages. It is refreshing to note that these movies have been well researched and psychiatrists are portrayed in a positive way. These kind of movies will augur well for the mental health profession and for psychiatrists as a whole.

There are no simple remedies to tackle this menace. It is important for all of us, as psychiatrists, to put a barrier to this media bashing. It is our responsibility to create more awareness in the media about mental illness. For this, we should leave no stones unturned as the costs of neglecting this aspect can be profound. All of you must have seen celebrities like Amitabh Bachchan, Aishwarya Rai, Hrithick Roshan etc… appearing in the electronic and print media campaigning for various public health causes like polio, breast feeding, AIDS etc… Have you ever seen any celebrity endorsing psychiatry in which ever format….? I can't think of any. Imagine a day, when celebrities endorse mental illness and the impact that will have on the common man. I certainly feel that is a possibility and we in IPS are taking some initiatives to that extent. I urge all members of IPS to leave the “Media Shy” attitude and come out more willingly with a positive attitude and dispel the myths about psychiatry and do all that you can to enhance the image of psychiatry, which is required for the public to embrace our speciality.

## SUCCESS STORIES IN PSYCHIATRY

In this section, I would briefly highlight some of the defining success stories in the field of mental health. The ones that immediately come to my mind are the awe inspiring work done by the psychiatrists in the aftermath of the ‘Tsunami’ tragedy. The work has brought a lot of recognition for psychiatry and psychiatrists. This is particularly significant as this kind of work will go a long way in making the public perceive more closeness and trust in psychiatry. The extensive work done by psychiatrists during the recent farmers' and weavers' suicides and the so called ‘Banamati’ (witch craft) in the south Indian states of Andhra Pradesh and Karnataka is a case in point. These instances go a long way in increasing the closeness with the policy makers and the public alike. I also compliment the kind of training, research and clinical work that is going on in the premier central government institutes like NIMHANS, Bangalore and PGI, Chandigarh. I also praise the efforts of all the psychiatrists who have created awareness in the community about mental illness and strived hard to dispel the myths.

## VISION FOR PSYCHIATRY IN INDIA

Psychiatry must remain a holistic discipline with a bio-psychosocial approach as its model. That more than any other speciality, psychiatry looks at the totality of a human being. No other speciality of medicine has such a special understanding of human distress as psychiatry has. This understanding of human suffering, both medically and psycho-socially is our special double heritage and we must not give up one for the sake of the other. Mental health services must become more relevant for Indian cultural needs.

Medicine and psychiatry do not develop in a vacuum, but they develop in a historical social context. Various psychiatric terms, systems of diagnosis and classifications and approaches to management, are all based on European philosophical thoughts. This is not a very comfortable situation for a country like India, with its own rich philosophical heritage. Perhaps, no other civilization has considered understanding the functions of the human mind, psychopathology and the management of various mental disorders, the way we have in India. Yet, we continue to blindly follow alien concepts and methods, even though these are often inappropriate in our socio-cultural context. We need to deliberate on these issues and evolve a truly indigenous approach to mental health. Operational strategies derived from such an approach will accord more closely with ground realties, particularly in respect of psycho-social therapeutic interventions. India's biggest wealth is its human resources and therefore psychological well being of the community takes precedence for a faster growth and development of the nation and we as mental health professionals should facilitate that process.

## VISION FOR OUR NATION'S MENTAL HEALTH SYSTEM

- I believe every citizen of this country has a right to a comprehensive evaluation and an accurate diagnosis which leads to an appropriate, individualized plan of treatment.
- Patient and family centered, community based, culturally sensitive and easily accessible care without discriminatory administrative or financial barriers or obstacles
- Care in the least restrictive setting possible that encourages maximum independence
- Integration of mental health care with primary health care
- Research into the etiology and prevention of mental illness and into the ongoing development of safe and effective treatment interventions
- Combat and overcome the stigma historically associated with mental illness through enhanced public understanding and awareness
- Health benefits, access to effective services must be the same for people with mental illness as for other medical illnesses;
- Access to mental health care should be provided across numerous settings, including the workplace and schools
- Patients deserve to be treated with dignity and respect
- More resources should be devoted to treatment and to training an adequate supply of psychiatrists, to meet the current and future needs of the population

I took psychiatry as my profession three decades ago with a lot of passion and interest. I am very happy professionally. But I do feel disturbed to note the way in which psychiatrists are undervalued in the society today. A day should come when we should be proud to say that “I am a Psychiatrist” and future leaders in medicine are psychiatrists and leaders are joining psychiatry. This will happen if we gear ourselves to offer solutions to a wide range of personal, family and pressing social issues like suicides in farmers, weavers and students; prevailing myths and the ghastly consequences of black magic in some parts of our country; role in disaster management (Tsunami, Earth Quakes, Terror Attacks etc…). We should make the individuals acknowledge that their emotional health deserves the same careful attention given to their physical health. Focus should be on creating long term solutions for the entire population rather than looking at a narrow segment of only those who are extremely dysfunctional and ill. We should make the community realize that, what we are is what our minds are. Our bodies are merely the structures which house the mind. To understand this fully, consider the patient with severe Alzheimer's disease; without a mind, the body is a mere shell. If the mind is so important to us, it is important that we protect it to the maximum extent possible. Why is the medical profession geared to protecting the outer shell with such little emphasis laid on the valuables within? In this context, consider Stephen Hawkins, who holds the Lucasian Chair of Physics (once occupied by Isaac Newton!) in the UK. Hawkings' body is totally paralyzed by amyotrophic lateral sclerosis and he has to use a voice synthesizer even to speak; yet, look at the greatness of the mind within and all that it has achieved.

I imagine a day not in too distant future when people might make appointments for “Psychological Check Ups” like general health check ups. We need to be visionaries for the mental health needs of the entire community and not only the service providers for sick individuals. In these days of Gandhigiri, I would like to quote the Mahatma, “Be the change that you want to see in the world”. If we play our role as responsible psychiatrists, I envision a day, when psychiatry in general and psychiatrists in particular would receive their rightful place and become a household word. My appeal to all of you is that each one of you needs to participate in fulfilling this mission of “Making Psychiatry a household word”

Thank you, one and all

Long live IPS.

## References

[1] Murray CJL, Lopez AD (1996). The global burden of disease and injury series, volume 1: A comprehensive assessment of mortality and disability from diseases, injuries and risk factors in 1990 is and projected to 2020.

[2] Penn DL, Martin J (1998). The stigma of severe mental illness: Some potential solutions for recalcitrant problem. Psychiatr Q.

[3] Corrigan PW, Penn DL (1999). Lessons from social psychology on discrediting psychiatric stigma. Am Psychol.

[4] Link BG, Struening EL, Rahav M, Phelan JC, Nuttbrock L (1997). On stigma and itsconsequences: Evidence from a longitudinal study of men with dual diagnoses of mental illness and substance abuse. J Health Soc Behav.

[5] Eronen M, Angermeyer MC, Schulze B (1998). The psychiatric epidemiology of violent behaviour. Soc Psychiatry Psychiatr Epidemiol.

[6] Elbogen EB, Van Dorn RA, Swanson JW, Swartz MS, Monahan J (2006). Treatment engagement and violence risk in mental disorders. Br J Psychiatry.

[7] Regier DA, Narrow WE, Rae DS, Manderscheid RW, Locke BZ, Goodwin FK (1993). The de facto US mental and addictive disorders service system. Epidemiologic catchment area prospective 1-year prevalence rates of disorders and services. Arch Gen Psychiatry.

[8] Kendler KS, Gallagher TJ, Abelson JM, Kessler RC (1996). Lifetime prevalence, demographic risk factors and diagnostic validity of nonaffective psychosis as assessed in a US community sample. The National Comorbidity Survey. Arch Gen Psychiatry.

[9] Hanson KW (1998). Public opinion and the mental health parity debate: Lessons from the survey literature. Psychiatr Serv.

[10] Grob GN (1991). The severely and chronically mentally ill in America: Retrospect and prospect. Trans Stud Coll Physicians Phila.

[11] Thompson TL, Folks DG, Silverman JJ (1997). Challenges and opportunities for consultation-liaison psychiatry in the managed care environment. Psychosomatics.

[12] Vir Sanghvi (2006). Campaign against Sect. 377. Editorial, Hindustan Times.

